# Spurious Pregnancy

**Published:** 1893-08

**Authors:** Bruce P. McVey

**Affiliations:** Mumford, Texas


For Texas Medical Journal.

### SPURIOUS PREGNANCY.

BY BRUCE P. M'VEY, M. D., MUMFORD, TEXAS.

THE case I am going to tell you about was not a case of spurious pregnancy simple, but that of a supposed true pregnancy in the beginning, followed by abortion at two months, ascites, etc.

Martha U., colored, aged about 40, consulted me last December, to know why her baby had not been born. Said she was then eleven months gone, and as yet no baby had shown up, and being the mother of fifteen children, she knew there was something wrong.

I scanned her contour, and said to myself, this is either a protracted pregnancy or an error in dates. She went on to tell me that since a few months after the birth of her last child, her menses had been perfectly regular till about eleven months previous, when they stopped suddenly. She suffered no inconvenience from this, however, and supposed herself in the family way, but owing to some heavy lifting about the end of the second month, she tried to abort, having labor pains and hemorrhage. She summoned a physician in time, and was soon all right; but lo! at the end of the third month her menses came again, and she discovered her abdomen growing soon after, and matters thus continued to the time when she consulted me; her menses coming on regularly and her abdomen still growing.

Seeing her as she was, and hearing her talk so sensibly and straight about it, naturally made me open my eyes. So I questioned her further, and she told me that she could feel the child move distinctly, and at the ninth month from the time her courses first stopped, she was in labor a day and night, and had a midwife with her, but matters failed to materialize.

I examined her, and found her abdomen about the size of a nine month's pregnancy, perfectly symmetrical, and soft, as though the whole contents were liquor amnii; but never having thumped a pregnant woman for ascites, I neglected it, and thus erred in my diagnosis, or rather, failed to come to any satisfactory conclusion.

You may say that a digital examination and the fact of her having had her courses, ought to have cleared up matters, and perhaps it ought, but it didn't. The cervix was large and soft, but not overly high up, neither did it have that same feeling, that you all know better than I can describe, that you feel in a woman about to bring forth. Balottement gave no indications of pregnancy.

I recalled to mind cases that I have read about, where menstruation continued clear through the period of gestation, and a case of my own, where the woman, a multipara, was in the habit of menstruating to the fifth month of her pregnancy.

The best idea that I could arrive at was that the child had died in utero when small, and the liquor amnii continued to accumulate. I had her former physician, Dr. C. D. Cearnal, who was close by, summoned. He said that he attended her when she was trying to abort, and that she was having labor pains and hemorrhage. He prescribed appropriate remedies, and soon controlled the pain and hemorrhage, but whether she succeeded in aborting or not, he could not say. I then asked him to examine her, and he, having a physician's eye not blinded by obstetrical zeal, thumped her abdomen once or twice, and told me that in his opinion the enlargement was dropsical, and after a thought and a thump on my part, I most readily coincided with the diagnosis. We prescribed Basham's Mixture, and told her that she was not pregnant, and in a few weeks she was apparently in first rate health. She has not been pregnant since, neither had dropsy. There was no question about the enlargement being due to an accumulation of fluid.

Now, dear reader, you have heard my story, and I beg that you be as honest as I have tried to be, and tell me, under the circumstances might not you have been bothered in a diagnosis, too? I know a number of you would not, while many more will say you would not. A multiplicity of contradictory symptoms caused me to entertain, temporarily, erroneous ideas. Now, after having read this, may it not be your lot to be similarly misled, but I would say to you, always keep in mind that every big belly doesn't contain a baby, even if it is on a woman.

